# Supplementary material for: Effects of Blanching, Freezing and Canning on the Carbohydrates in Sweet Corn
Source: Foods. 2023 Oct 24;12(21):3885. doi: 10.3390/foods12213885 (PMC10649117; doi:10.3390/foods12213885)

Supplemental Table S1. Results of quality control material assessment.

Component	QC Material	Certificate of Analysis [a]		Target Values [b]		Assayed	
		Target - Uncertainty	Target + Uncertainty	Mean ± SD	Range	Batch 1	Batch 2
Glucose (g/100g)	SRM® 3233 Breakfast Cereal	0.7	1.4			0.9	0.9
Sucrose (g/100g)	SRM® 3233 Breakfast Cereal	9.6	17.2			13.6	13.8
Fructose (g/100g)	SRM® 3233 Breakfast Cereal	0.4	1.2			0.8	0.7
IDF (g/100g)	SRM® 3233 Breakfast Cereal	5.3	7.9			5.8	5.9
HMWDF (g/100g)	SRM® 3233 Breakfast Cereal	6.4	12.0			9.2	9.5
LMWDF (g/100g)	SRM® 3233 Breakfast Cereal	1.8	4.2			2.0	2.2
TDF (g/100g)	SRM® 3233 Breakfast Cereal	9.2	14.6			11.2	11.7
Starch (g/100g)	flour QC material			55.2 ± 3.3	45.8-58.9	56.1	55.4

[a] For commercial reference materials, reference range from certificates of analysis.

[b] Targe values (including Mean ± SD and range) were obtained after 27 runs on the QC material.

Supplemental Figure S1. PCA loading plot of the glycosidic linkages of total polysaccharides.

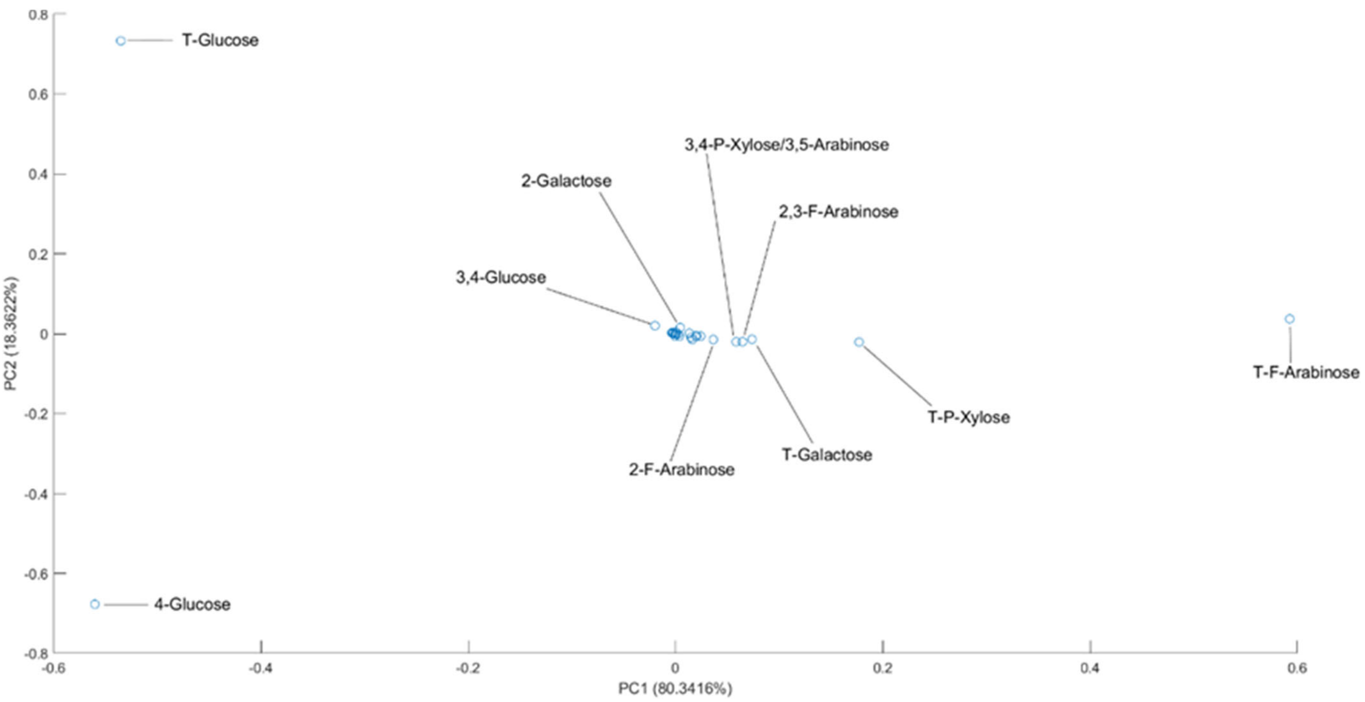

Supplement: Supplementary file 1 [file foods-12-03885-s001.zip › foods-2659418-supplementary.pdf]
